# Does supplier concentration matter to investors during the COVID-19 crisis: evidence from China?

**DOI:** 10.1186/s40854-022-00391-0

**Published:** 2022-09-26

**Authors:** Louis T. W. Cheng, Jack S. C. Poon, Shaolong Tang, Jacqueline Wenjie Wang

**Affiliations:** 1 School of Business, The Hang Seng University of Hong Kong, Hong Kong, China; 2 School of Accounting and Finance of the Hong Kong Polytechnic University, Hong Kong, China; 3 Division of Business and Management, Beijing Normal University-Hong Kong Baptist University, United International College, Zhuhai, China

**Keywords:** COVID-19, Supplier concentration, Supplier disclosure, Stock price effect, Industry neutral portfolio

## Abstract

The literature shows that investor attention to customer–supplier disclosure increases when suppliers’ information arrival is anticipated. Due to the widespread of city lockdowns in China and the implementation of social distancing to control the COVID-19 pandemic, investor attention to potential disruption of the supply chain spikes, leading to a price devaluation for firms with high supplier concentration risk. We find that a higher degree of supplier concentration is related to more serious stock price declines over the short-term and medium-term windows right after the Wuhan lockdown. This result lends support to the argument that the concentration risk of suppliers is a significant consideration for China stock market investors, especially under the potential financial distress at the firm level induced by the COVID-19 crisis.

## Introduction

Supply chain viability and resilience have been one of the most important issues for firms to ensure the sustainability of business operations (Ivanov and Dolgui 2020; Hosseini et al. 2019). The outbreak of COVID-19 is believed to exert stress to the supply chain and lead to serious operational and financial risks to firms globally (Ivanov and Das 2020; Ivanov 2020). As a result, the literature on supply chain viability and survivability under unexpected global disruption such as the COVID-19 crisis becomes very important to both the academic world and industry practitioners. Therefore, a closer examination of the financial consequences of supply chain deterioration due to the city shutdowns in Mainland China through stock price reaction can provide insight into the ripple effects of supply chain concentration under external shocks (Dolgui et al. 2018; Ivanov 2018).

The pandemic was originated in the form of a health crisis. However, due to the implementation of social distancing policy and subsequent shutdown of many cities as a stop-gap measure to fight the virus, it has quickly turned into an economic crisis with various business implications for academic research. For instance, Youssef et al. (2021) demonstrate that there is a clear dynamic connectedness among eight stock markets and the effects of economic policy uncertainty during the COVID-19 pandemic. One obvious and important research question related to the Wuhan lockdown is the stock market investors’ expectation and possible negative reaction to the supply chain deterioration of Mainland Chinese firms when the China stock market opens right after the Wuhan lockdown.

The COVID-19 had accumulated a series of bad news over a period since the first symptoms appeared during December 2019. The first death and clinically confirmed infection were reported on 10 January 2020 before the announcement of Wuhan lockdown on 23 January 2020. In April 2020, Fung Business Intelligence reported^1^ that ‘the COVID-19 outbreak would cause more adverse and extensive disruptions to the Chinese economy compared with the SARS outbreak in 2003’. It was not only due to extensive geographical areas being affected but also the tough containment measures imposed by the Chinese government in order to contain the pandemic. The report also stated that during the COVID-19 outbreak between January and February 2020, industrial production, fixed-asset investment, retail sales, and exports had dropped by 13.5%, 24.5%, 20.5%, and 17.2% respectively. An important measure for the prevailing direction of economic trends in manufacturing, the Purchasing Manager Index (PMI), fell from 50.2 in December 2019 to 37.7 in February 2020. Many countries implemented stringent virus containment and prevention measure. Across the world, there was large-scale production suspension, logistic disruption across borders and within countries, and widespread demand-side disruption due to order cancellation.

**Fig. 1:**
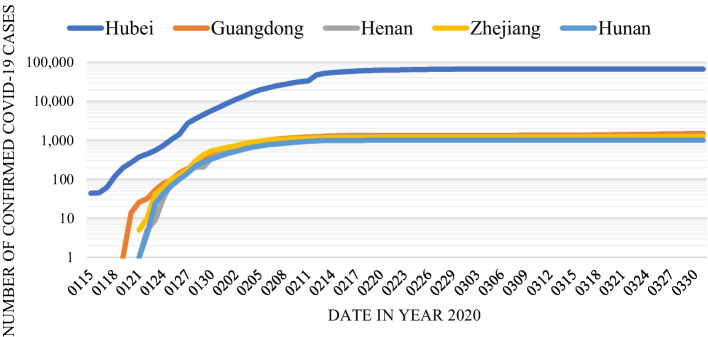
Number of confirmed COVID-19 cases in China's Top 5 Provinces. Source: China Data Lab, 2020, "China COVID-19 Daily Cases with Basemap". 10.7910/DVN/MR5IJN, Harvard Dataverse, V32.

**Fig. 2:**
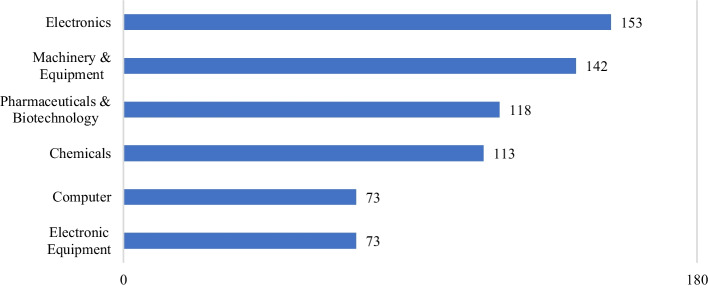
Top 6 Industries of A-Share Listed Firms in the 5 Worst-Hit Provinces (Guangzhou, Zhejiang, Henan, Hubei, Hunan). Source: “Covid-19 Impact on China A-Shares' Supply Chains” V1.0 February 2020, MioTech

To illustrate the extent of such adverse effects due to the supply chain deterioration, Fig. 1,^2^ shows the confirmed infected cases for the worst five provinces in China, namely Hubei, Zhejiang, Guangdong, Henan, and Hunan. This figure depicts a sharp arising pattern from around late January to early February 2020. Then the number of confirmed cases maintains a steady pattern throughout the rest of our studied period. Corresponding to the outbreak in these five provinces, Fig. 2 shows the number of listed firms in the top six industries located in these five hardest-hit provinces as reported by MioTech.^3^ These six industries are Electronic Equipment (73 firms), Computer (73 firms), Chemicals (113 firms), Pharmaceuticals & Biotechnology (118 firms), Machinery & Equipment (142 firms), and Electronics (153 firms). These numbers reflect the pervasiveness and scope of the potential supply chain shock in early February. Therefore, we expect a market-wide devaluation in the China stock market when it opens for trading after the Chinese New Year holidays on 3 February 2020.

Specifically, we expect that the lockdowns of various cities in China would generate a substantial supply chain disruption in the manufacturing sector, especially with industry related to manufacturing.^4^ Consequently, this supply chain shock due to COVID-19 would severely affect the liquidity and cash conversion cycle of a firm. Without sufficient alternative suppliers to maintain production or operation, firms may face serious disruption and loss of revenue. In short, supply chain disruption due to COVID-19 and the countrywide city lockdowns in China can result in a sharp reduction of cash flow and an increase in firm-level business risk. Thus, these series of events may lead to a firm’s stock price devaluation. China is a global manufacturing hub and the first country to implement city lockdown. Examining the first opportunity of stock price reaction to Chinese firms with different supplier concentrations may reveal a better understanding of how investors evaluate supplier concentration risk under severe shocks, such as the COVID-19 pandemic. Our findings will shed light on listed firms globally for their supply chain risk management and will possibly provide a remedy to fight the financial distress from a future pandemic.

The accumulation of negative news leading to the Wuhan lockdown may imply a stronger impact on firms with higher supplier concentration. As a concentrated relationship in the supply chain limits the sources of money flow between suppliers and customers, a firm’s ability to pay back its debt and the pricing of borrowing costs would be negatively affected. In this study, we postulate a negative relationship between supplier concentration and stock price reaction after the negative news shock from the Wuhan lockdown. Specifically, we examine the short-term and medium-term stock returns right after the news of the Wuhan lockdown when the China stock market opens after the Chinese New Year holidays. Using the CSI300 index as a proxy for the stock price broad reaction for China stock market, the market did not initially realize the seriousness of the downside risk prior to the Wuhan lockdown.^5^ Immediately after the announcement of the Wuhan lockdown on 23 January 2020, the China stock market was closed the following day for the Chinese New Year holidays. When the market opened after the holidays on 3 February 2020, the CSI300 index dropped sharply from 4131.93 (closing on 22 January 2020) to 3688.36 (closing on 3 February 2020). By March, COVID-19 became a global pandemic. Globally, multiple stock markets experienced sharp declines, and the CSI300 index dropped to the lowest point at 3530.31 on the closing of 23 March 2020. We aim to capture the initial market response to the negative price effect of the supply chain deterioration of the Chinese firms, which is one of the most important concerns from the stock market investors. Investor’s attention on the valuation of information arrival intensifies in the context of customer–supplier disclosure (Madsen 2017).

The literature has documented that there are advantages and disadvantages to the concentration effect. When an economic crisis such as the COVID-19 pandemic occurs, a firm with highly concentrated suppliers may potentially become a huge risk leading to business disruption due to a broken supply chain. We conjecture that there exists a significant initial market response to the negative price effect of the supply chain deterioration of Chinese firms. Our theoretical support comes from Madsen (2017), which demonstrates a linkage between anticipated information arrival and investor attention in the context of customer–supplier disclosure. Under the COVID-19 induced financial distress, we expect a similar linkage between investor attention and public disclosure of suppliers’ information, leading to a price devaluation due to the potential disruption of the supply chain as a result of the Wuhan lockdown.^6^ Specifically, we argue that the lockdowns of various cities all over China and social distancing would increase investor attention to the supplier disclosure and concentration. Such investor attention may lead to a downward stock price adjustment to reflect the supply chain disruption to Chinese firms. Our analysis shows that supplier concentration can significantly affect stock returns over the short-term windows (i.e. [− 1, 1] and [− 2, 2]) and a medium-term window [− 2, 100] around the Wuhan lockdown. Specifically, the higher degree of supplier concentration has a bigger stock price decline during the COVID-19 pandemic.

Our contribution can be summarized as follows. Based on the current global phenomenon and wide-coverage from the media,^7^ there is no question that COVID-19 disrupts global supply chain, resulting in shortage of all kinds of goods and industrial parts essential for the production process. Mainland China is the first country suffering from the supply chain disruption due to the lock down of cities as a result of COVID-19. Our paper is one of the earlier efforts to explore how concentrated suppliers may negatively affect stock valuation caused by a potential supply chain deterioration. In short, during the COVID-19 pandemic, supplier chain deterioration is an important issue. Since there is no requirement on disclosure of suppliers in the US, prior studies using US listed firms employ reverse disclosure to collect data on suppliers. However, the advantage of examining firms listed in China is that they are required to list top 5 suppliers in their annual reports, which could allow us to examine supply chain disruption and its effects on stock valuation during crisis. This is our first contribution.

In addition, we also contribute to the literature by employing investor attention argument by Madsen (2017) to capture suppliers’ information arrival. We show that the corresponding investor attention to this negative supply chain information leads to a price devaluation for firms with high supplier concentration risk. Our study provides insight for top management to seriously consider supplier diversification to prevent unexpected supply chain disruption. In addition, our study indicates that, during the normal period, lower supply concentration can generate superior returns for an investment portfolio. More importantly, lower supplier concentration can also reduce stock price decline during unexpected nationwide crisis.

## Literature review and hypothesis

While our study focuses on the stock price effect of supply chain disruption related to supplier concentration under COVID-19, it is useful to briefly review the general literature on supply chain viability and its ripple effects under economic difficulties. Emtehani et al. (2021) argue that, under economic recession, an effective coordination of the supply chain through a joint decision-making on the physical and financial flows of a capital-constrained supply chain model can help firms to stay competitive and maintain market share.

Dolgui et al. (2018) provide a comprehensive review of the supply chain ripple effect literature. They define ripple effect as the impact of disruption propagation on supply chain performance and disruption-induced changes in supply chain parameters. The paper also presents the ripple effect control framework that includes redundancy, flexibility, and resilience analysis. For countermeasures, they recommend geographical sourcing diversification to avoid delay in disruption recovery. Risk mitigation is an important aspect of achieving supply chain viability. Yoon et al. (2018) evaluate the efficacy of alternative risk mitigation strategies and recommend both upstream and downstream solutions should be employed simultaneously. Choi et al. (2020) employ an innovative approach through game theory applications under the context of sharing and circular economy, providing a possible solution for businesses that treasure environmental sustainability. Most recently, Ivanov and Dolgui (2020) and Ivanov and Das (2020) evaluate supply chain resilience under the COVID-19 crisis and provide new perspectives for risk mitigation and recovery paths.

In terms of related benefits for supplier concentration, various operation management studies have examined how sharing of supply chain information would improve operational efficiency and reduce operation cost (Bourland et al. 1996; Cachon and Fisher 2000). Studies of supply chain visibility have definitely shown that improvement through sharing high-quality information and creating a tight linkage between suppliers and focal firms would lead to operational improvement and cost reduction in the supply chain. Ak and Patatoukas (2016) find evidence of a valuation premium for high customer-base concentration. Investors trade off the costs and benefits of relationships believing that firms with higher concentration hold significantly fewer inventories and experience shorter inventory holding periods.

Concentrated suppliers may lead to over-dependency on suppliers and weakening of bargaining power. This may result in purchasing price increase and high switching costs when a supplier relationship is terminated. Early researches on supplier concentration focus on audit services because of the availability of publicly disclosed information and the high levels of auditor concentration. Interestingly, the research finds that audit fees are negatively related to industry concentration (Pearson and Trompeter 1994). Such finding is confirmed by later research on a small and private segment of the audit market where increased concentration does not necessarily lead to decreased price competition but rather to increased price competition (Willekens and Achmadi 2003).

On the other hand, we draw a similarity on financial effect between customer concentration and supplier concentration. Campello and Gao (2017) find that higher customer concentration generally increases the interest rates and the number of restrictive covenants on bank loans. This shows that the bank sees a higher risk for concentration, thus, an interest rate premium is applied to compensate for the concentration risk associated with the customer. Sun and Li (2018) study the impact of concentration on bond credit spreads for over 700 Chinese firms between 2009 and 2016. The research concludes that bond investors translate high supplier concentration to a higher risk premium. These two pieces of researches indicate that higher concentration either in customers or suppliers translates to a higher risk premium reflected by the borrowing cost. Zhang et al. (2020) examine over 2000 Chinese firms to evaluate the impact of supplier concentration on a firm’s cash holding between 2009 and 2016. This research concludes that a firm’s cash holding is positively associated with supplier concentration. The finding can be explained by the weakening of bargaining power when there is a strong dependency on a supplier. As a result, the firm experiences a decline of the firm’s trade credit, hence, holding more cash for precautionary consideration.

Madsen (2017) finds that attention to a firm’s publicly disclosed customers increases before the firm announces earnings. It shows a linkage between anticipated information arrival and investor attention in the context of customer–supplier disclosure. This linkage is incorporated into price discovery and valuation by the market. Various operation management studies have examined how the sharing of supply chain information would improve operational efficiency and reduce operating costs.

In addition, recent articles have reported that COVID-19 has caused a major supply chain disruption globally. For instance, the National Health Service in the UK stated that COVID-19 has created the “Great Supply Chain Disruption” (Goodman and Bradsher 2021). The US White House issued an article on 17 June 2021 to explain why the pandemic has disrupted supply chains in the US (Helper and Soltas 2021). In May 2020, PwC China released the finding of surveys conducted in October 2019 and March 2020^8^ to study the supply chain impact on US companies operating in China. Overall, the result shows that COVID-19 limits operations below normal capacity but over 70% of companies have no plans to relocate supply chain operations outside China due to COVID-19. In short, these articles have suggested that the supply chain disruption has impacted normal lives globally but no obvious indication to shifting suppliers outside of China by foreign firms. Therefore, the pandemic has indeed caused a serious supply chain disruption.

Based on the above literature, we conjecture that suppliers’ concentration would induce additional financial and operational risk to the firm under the COVID-19 pandemic. This may result in a stronger downward price adjustment to the listed firm when the stock market opens right after the Chinese New Year holidays after the Wuhan lockdown. Our argument of relating the potential supply chain deterioration and stock market reaction is based on Madsen (2017), which shows that investor attention to customer–supplier disclosure increases when suppliers’ information arrival is anticipated.

In our setting, the information arrival refers to the COVID-19 seriousness and the corresponding evaluation from the investors based on the supplier concentration as shown in the previous financial statement. While the supplier concentration information has already been disclosed in previous financial statements, this information related to the conclusion of higher risk of supply chain disruption does not exist during normal business environment and economic conditions without COVID-19. In other words, even though the financial statement has already disclosed the supplier concentration information, the COVID-19 pandemic is new. Notice that the stock market was closed for a long period of time during the prolonged Chinese New Year holiday while the COVID-19 infected cases kept increasing. The pandemic quickly turned into a serious lockdown and negative news continue to hit the investors. Thus, the investors quickly developed anticipation of the information arrival and its possible negative effect on firms with high supplier concentration. In short, the investor recognition of the additional supply chain disruption as a result of the interaction between supply chain concentration and COVID-19 is new to the market and leads to a negative stock valuation effect.

In fact, the investors cannot react to this news immediately as the stock market was closed during the lockdown period. Therefore, the anticipation of this new information related to the negative effect on the business operation and profitability of firms with high supplier concentration becomes relevant after the stock market reopened. In short, our event window is the first moment that the investor’s attention and the corresponding valuation assessment due to the linkage of COVID-19 and suppliers’ concentration can be reflected in the stock market, which is a new information arrival.

Such a linkage between suppliers’ information and corresponding stock return is consistent with our expectation that investor attention to potential disruption of the supply chain would increase. In sum, we hypothesize that due to the widespread city lockdowns in China and the implementation of social distancing to control the pandemic, investor attention spikes, and the market turns its attention to the supplier disclosure and concentration to evaluate the potential damage on stock valuation for firms in China. Consequently, investors turn their attention to the public disclosure of suppliers’ information, leading to a price devaluation for firms with high supplier concentration risk.

**Hypothesis** Stock returns around the Wuhan lockdown is negatively related to supplier concentration.

## Data and methodology

We chose Chinese firms for our analysis based on two reasons. First, the COVID-19 crisis began in the city of Wuhan in China. The world was watching closely how the COVID-19 pandemic is translating into financial impact when the China stock market opened after the Chinese New Year holidays after the Wuhan lockdown. It is important to study how the linkage of investor attention to price discovery due to the supply chain disruption. Second, the availability of the supplier’s data allowed a more comprehensive analysis. Chinese regulation mandates listed firms to disclose the top 5 suppliers’ spending amounts in their annual report. Contrarily, the US has no regulation on mandatory supplier disclosure, and firms choose to disclose the information on a voluntary basis. Hence, the data from the Chinese firms is the only feasible and logical choice to examine the price effect of supplier disclosure under the COVID-19 pandemic.

### Supply chain data construction

The supply chain dataset was obtained from MioTech, a fintech startup that uses artificial intelligence (AI) to collect public data, mainly in the area of supply chain and ESG. In the dataset used for this research. MioTech’s AI engine extracts disclosed information directly from the firm’s annual report. As CSMAR also provides data for the top 5 suppliers, which is a mandatory requirement by the China Securities Regulatory Commission (CSRC), it is important for us to explain why we use MioTech’s data instead. The major reason to employ MioTech’s data is their inclusion of ‘reverse’ disclosure to strengthen the supply chain data set through their AI engine.

Our initial dataset contains the disclosed suppliers’ information between 2016 and 2019 for 3,700 A-share firms that are publicly listed in China’s stock exchanges. China’s public disclosure requires listed firms to disclose the purchase amount and percentage of total purchase from at least the top 5 suppliers. The supplier’s name can be anonymized.

While we started with a sample of 3700 firms, two major reasons cause a substantial drop in valid firms for analysis. Owing to COVID-19, many listed firms could not complete their audit annual reports as expected for the year of 2019. Social distancing policy leads to office shutdowns and Chinese firms were not prepared to have the accounting and finance office to function at home office. Consequently, suppliers’ data in audited reports were not available till many months later. At the time of our data purchase in early 2020, 1073 listed firms had not reported the suppliers’ data, which substantially reduced our sample size. In other years, some firms simply did not file the data due to unknown reasons. In addition, 61 firms filed the names of suppliers but did not disclose the transaction amount. Finally, our sample size is reduced to 2062 as reported in Table 1. The sample size for 2019 was 1965 firms after removing 97 financial and utility firms.^9^

**Table 1:** Sample size reduction

Summary	Count
Original Dataset	3700
Company without any suppliers’ data 2019	− 1073
Company without any suppliers’ data 2018	− 186
Company without any suppliers’ data 2017	− 87
Company without any suppliers’ data 2016	− 231
Company without 2019 supplier disclosed amount (2016–19)	− 61
Total*	2062

*The sample size for 2019 was 1965 firms after further removing 97 financial and utility firms

In this study, we adopt the dataset from MioTech, which employs an AI engine to extract suppliers information using both forward disclosure and reverse disclosure approaches to enhance the data for our sample firms. Forward disclosure is the regular disclosure captured from the annual reports of the focus firms (CSMAR uses this approach to construct its dataset). The forward disclosure reported in the annual reports capture the top 5 suppliers as required by CSRC.

The method of “reverse disclosure” in supply chain studies is a common procedure used by US studies. In the US, no mandatory supply chain disclosure for top-5 suppliers is required by the SFC. US firms are required to disclosed transaction amounts and names of customer of 10% or higher of total transaction value. Therefore, US studies (e.g., Patatoukas 2012; Zhao et al. 2019), have to employ indirect method to back-track suppliers’ information from customers’ disclosure to complete the suppliers’ information of the focus firms for their empirical analysis. This indirect method is being labelled as “reverse disclosure” by academic researchers (borrowed from the concept of reverse engineering) and also by MioTech. When the MioTech AI engine enhanced the data with reverse disclosure, the engine checks all the overlapping disclosure using both methods and we manually double checked the combined file to make sure that we do not include duplicated information. In our sample, 114 firms in 2019 and 166 firms in 2018 contain additional supplier information through reverse disclosure. Finally, as MioTech adopts SWS industry classification which is believed to better define supply chain industry groups, we follow the SWS classification for our regression analysis.

### Forward data and reverse data

First, firms from these three industries, Public Utilities, Banking, and Non-bank financial industry, are excluded from our sample. Second, some firms are further removed due to missing data. Next, the forward list and reverse disclosure are combined and sorted based on the purchase amount in order to extract the top 5 suppliers. Since the data set from MioTech does not provide the total supplier’s contracted amount of a firm, which is needed to generate the supplier disclosure and subsequently concentration ratio in percentage, our first task is to compute the total supplier amount.

For the reverse disclosure, the supplier can disclose its revenue and percentage of total revenue to a particular customer, but it falls short of disclosing how much its revenue constitutes as a percentage of its customer’s total supplier spending. Hence, in the case of reverse disclosure, the percentage of the total supplier amount must be computed based on the forward disclosure’s information. Specifically, the total supplier amount is computed with the following formula in the forward disclosure:

1 $$Total Supplier Amount= \frac{\sum_{i=1}^{N}{Supplier Amount}_{i}}{\sum_{i=1}^{N}{Percentage of Supplier Amount}_{i}}$$

The total supplier amount can be derived using one supplier’s purchasing amount and its percentage. Using the summation of both the numerators and the denominators can average out the rounding effect caused by an individual supplier’s calculation. After computing the percentage of supplier amount in both forward and reverse disclosure, the supply amount and the name for the top 5 suppliers are extracted for 2018 and 2019.^10^ We define two variables using the top 5 suppliers, i.e. Supplier Disclosure Index (SI) and Supplier Concentration (SC). The two variables *SI* and *SC* use both forward and reverse disclosure.

2 $$SI = P_{F} + P_{R} ,$$

SI is the combined percentage of supplier spending amount from forward and reverse disclosure (*P*_F_ and *P*_*R*_). When the *SI* percentage is high, investors and analysts know more (relative to the total transaction amount) about where the supplies come from, which can be useful information for evaluating supply chain risk and the corresponding valuation.

3 $$SC=\sum_{j=1}^{J}{\left(\frac{{Supply}_{j}}{Total supply amount}\right)}^{2},$$

In order to conduct regression analysis, the literature use another variable to measure supplier concentration, “SC”. SC uses the same information as SI but it is constructed based on the format of Herfindahl–Hirschman Index, which is a very common measure for market concentration in economics. This method is also commonly used in supply chain literature (e.g. Patatoukas 2012; Ak and Patatoukas 2016). ${Supply}_{i}$ represents a firm’s supply spending amount from top 5 supplier *j* in a certain year using both forward and reverse disclosure data, *Total supply amount* represents the firm’s total supply amount in the same year, and *J* is the total number of major suppliers disclosed in the firm’s annual report, which is 5 in our case. The SC measure captures two elements of supplier diversification, i.e. the number of major suppliers with which the firm interacts, and the relative importance of each major supplier in the firm’s annual total supply. The range of SC should be between 0 and 1, where lower (higher) values correspond to less (more) concentrated supply. Table 2 shows the descriptive statistics for SI in decile. SI represents the sum of the top 5 suppliers’ spending amount over the total amount of supplier spending. The mean and standard deviation of SI for the 1965 firms are 32.7 and 20.1 respectively. Over 90% of the firms have SI above 10%. The sample shows a skew below the mean with over 85% of the firms fall between 0.7 and 0.1. Table 3 shows the descriptive statistics for SC in 12 uneven groups due to the quadratic nature of SC value. For values between 0.1 and 0, the statistics are described in 10 even quantiles. For values above 0.1, the statistics are divided into two quantiles with values between 0.1 and 0.2, and between 0.2 and 1.0. The mean and standard deviation of SC for 1965 firms are 0.051 and 0.090 respectively. Over 68% of the firms have SC above 0.01. The sample shows a skew below the mean with over 54% of the firms fall between 0.01 and 0.10.

**Table 2:** SI ratio statistics. Columns (1)-(6) report number of observations, means, standard deviations, medians, minimum and maximum of variable SI

Statistics	N	Mean	Std	Median	Min	Max
90% < = SI < 100%	18	93.962	2.917	93.115	90.490	100
80% < = SI < 90%	42	84.252	2.677	83.719	80.370	89.630
70% < = SI < 80%	48	75.012	2.732	75.550	70.350	79.680
60% < = SI < 70%	113	64.675	3.141	64.140	60.020	69.910
50% < = SI < 60%	151	54.657	2.850	54.250	50.055	59.930
40% < = SI < 50%	224	44.948	2.815	45.125	40.041	49.990
30% < = SI < 40%	329	34.752	3.024	34.530	30.020	39.980
20% < = SI < 30%	467	24.772	2.912	24.642	20.070	29.980
10% < = SI < 20%	390	15.352	2.775	15.320	10.007	19.990
0% < = SI < 10%	183	4.571	3.486	4.865	0.114	9.88
Total	1965	32.715	20.144	28.617	0.114	100

**Table 3:** SC statistics. Columns (1)-(6) report number of observations, means, standard deviations, medians, minimum and maximum of variable SC

Statistics	N	Mean	Std	Median	Min	Max
0.20 < = SC < 1.00	104	0.352	0.164	0.286	0.202	0.891
0.10 < = SC < 0.20	164	0.140	0.028	0.134	0.100	0.199
0.09 < = SC < 0.10	34	0.095	0.003	0.094	0.091	0.100
0.08 < = SC < 0.09	50	0.085	0.003	0.084	0.080	0.090
0.07 < = SC < 0.08	43	0.075	0.003	0.075	0.070	0.080
0.06 < = SC < 0.07	64	0.065	0.003	0.065	0.060	0.070
0.05 < = SC < 0.06	82	0.055	0.003	0.055	0.050	0.060
0.04 < = SC < 0.05	95	0.045	0.003	0.044	0.040	0.050
0.03 < = SC < 0.04	137	0.035	0.003	0.034	0.030	0.040
0.02 < = SC < 0.03	216	0.025	0.003	0.024	0.020	0.030
0.01 < = SC < 0.02	351	0.015	0.003	0.014	0.010	0.020
0 < = SC < 0.01	625	0.004	0.003	0.004	0.000	0.010
Total	1965	0.051	0.090	0.020	0	0.891

To control for the potential influence of disclosure and concentration pattern due to industry-specific supply change practice, we also develop the industry median-adjusted alternatives. SI_adjusted is the median-adjusted SI by subtracting the median of SI of each industry from SI. SC_adjusted is the median-adjusted SC by subtracting the median of SC of each industry from SC.

### Financial data

We acquire firm-level financial data for 2019 from the CSMAR database and corresponding stock data from Refinitiv’s Datastream. There are 1965 Mainland A-share stocks with all relevant data in our sample. The China stock market was closed between 24 January and 2 February 2020. We define event day 0 as the first trading date when the China stock market opened on 3 February 2020. Our short-term event windows for examining stock returns are 23 January, 3 February, and 4 February as day-1, day 0, and day + 1 respectively. In terms of calendar time coverage, *R*[− 1,1] refers to cumulative raw returns (%) over the three-trading day window between 23 January and 4 February 2020, after the Wuhan lockdown. *R*[− 2,2] refers to cumulative raw returns (%) over the five-day window between 22 January and 5 February 2020.

We follow Broadstock et al. (2020) for selecting control variables for our regression models. LnAsset is the logarithm of total assets in RMB 10 billion. BM is the ratio of book value per share to the stock price per share. Leverage is the ratio of total liability to total assets. Cumulative Market-adjusted Returns, denoted by $R\mathrm{^{\prime}}$[− 1,1] and $R\mathrm{^{\prime}}$[− 2,2] for the event window between 23 January and 4 February 2020, and the event window between 22 January and 5 February 2020 respectively, are calculated by subtracting the market return from the raw return. Cumulative abnormal returns, denoted by $R\mathrm{^{\prime}}\mathrm{^{\prime}}$[− 1,1] and $R\mathrm{^{\prime}}\mathrm{^{\prime}}$[− 2,2] for the event window between 23 January and 4 February 2020, and the event window between 22 January and 5 February 2020 respectively, are calculated by subtracting the expected return based on Capital Asset Pricing Model (CAPM) from the raw return, while the beta estimation of CAPM is over 200 trading days, i.e. 4 March 2019–23 December 2019. All cumulative returns and *Leverage* are winsorized at 2% and 98%.

In this study, we follow the literature, which mainly focuses on firms in the secondary sector (with industries concentrated in manufacturing) from an economic perspective. Nevertheless, it may be informative to report our summary statistics and selective findings for all firms and by primary, secondary, and tertiary sectors. We list our variable definitions in Appendix A. 11 We exclude firms belonging to industries in public utilities, banking, and non-bank financial as a standard research practice for empirical analysis. In Table 4, we present the statistics for the total sample, primary sector, secondary sector, and tertiary sector, respectively. The size of the total sample including all three sectors is 1,965 during our studied period. The largest group is naturally the secondary sector with 1,558 firms. The primary (i.e., natural resources and raw material extraction) sector has only 140 firms and the tertiary sector (i.e., service providers) has 267 firms. In terms of averages, the secondary sector appears to have a smaller SI and SC relative to the other two sectors. This indicates that firms in the secondary sector, which has a higher concentration of manufacturing firms, have lower top 5 supplier percentages and lower supplier concentration. No outliers or irregularities are observed from the descriptive statistics among these industry groups.

**Table 4:** Summary statistics

Stats	N	Mean	Std	P25	P50	P75	Max
All firms							
R[− 1, 1]	1965	− 12.218	8.138	− 17.397	− 14.211	− 9.468	21.711
R[− 2, 2]	1965	− 9.544	9.673	− 15.398	− 12.150	− 7.273	31.796
SI SI_adjusted SC SC_adjusted LnAsset BM Leverage	1965 1965 1965 1965 1965 1965 1965	32.715 3.712 0.051 0.030 − 0.954 0.632 0.435	20.144 19.4484 0.090 0.089 1.219 0.245 0.204	18.160 − 10.149 0.008 − 0.012 − 1.802 0.458 0.277	28.617 0 0.020 0 − 1.093 0.631 0.421	44.800 14.790 0.056 0.032 − 0.283 0.807 0.578	100 73.440 0.891 0.877 4.123 1.442 0.949
Primary							
R[− 1, 1] R[− 2, 2]	140 140	− 13.807 − 11.804	5.333 6.302	− 17.769 − 15.546	− 14.286 − 13.278	− 11.185 − 9.415	11.862 18.278
SI SI_adjusted SC SC_adjusted LnAsset BM Leverage	140 140 140 140 140 140 140	36.847 3.482 0.069 0.038 − 0.630 0.662 0.466	21.731 20.447 0.105 0.102 1.230 0.257 0.201	21.900 − 12.517 0.011 − 0.014 − 1.378 0.469 0.327	33.462 0 0.028 0 − 0.779 0.708 0.468	50.967 17.246 0.079 0.050 0.250 0.868 0.597	92.350 56.930 0.776 0.728 3.305 1.284 0.949
Secondary							
R[− 1, 1] R[− 2, 2]	1558 1558	− 11.959 − 9.167	8.324 9.874	− 17.295 − 15.238	− 14.136 − 11.967	− 9.057 − 6.689	21.711 31.796
SI SI_adjusted SC SC_adjusted LnAsset BM Leverage	1558 1558 1558 1558 1558 1558 1558	31.900 3.396 0.047 0.027 − 1.069 0.614 0.419	19.334 18.747 0.085 0.085 1.125 0.233 0.197	18.250 − 10.017 0.008 − 0.011 − 1.871 0.451 0.265	27.997 0 0.019 0 − 1.183 0.612 0.411	43.370 13.550 0.052 0.029 − 0.452 0.781 0.558	100 73.440 0.891 0.877 3.825 1.321 0.949
Tertiary							
R[− 1, 1] R[− 2, 2]	267 267	− 12.894 − 10.558	8.150 9.738	− 18.031 − 16.190	− 14.583 − 12.876	− 10.345 − 9.097	21.711 31.796
SI SI_adjusted SC SC_adjusted LnAsset BM Leverage	267 267 267 267 267 267 267	35.308 5.674 0.065 0.041 − 0.453 0.721 0.506	23.274 22.865 0.103 0.102 1.561 0.285 0.229	16.580 − 9.800 0.007 − 0.013 − 1.387 0.526 0.331	23.900 0 0.025 0 − 0.567 0.740 0.512	49.510 20.030 0.073 0.047 0.361 0.961 0.693	95.216 69.414 0.722 0.689 4.123 1.442 0.949

This table reports the mean (Mean), standard deviation (Std), median (Median), minimum (Min), 25th percentiles (P25), 50th percentiles (P50), 75th percentiles (P75) and maximum (Max) of stock return, supply information during the 2020 COVID-19 pandemic period, and other control variables. LnAsset is logged value of total asset in RMB 10 billion

### Regression model

To test our hypothesis, we define two alternative independent variables, namely SI_adjusted and SC_adjusted, to measure the degree of supplier concentration.^12^ Other control variables such as firm size, the book to market ratio, and the leverage ratio are included. The dependent variable is the cumulative return, which is measured by cumulative raw return, cumulative market-adjusted return, and cumulative abnormal return. To control for industry effects, we include 25 industry dummies defined by the sector code from SWS Research. We further add location fixed effects in all the tests and standard errors are adjusted for clustering at the industry level (in all tables including appendices).^13^

The empirical specifications of our multivariate regressions are:

4 $$R=\alpha +{\beta }_{1}SI\_adjusted+{\beta }_{2}LnAsset+{\beta }_{3}BM+{\beta }_{1}Leverage+{\sum }_{j}{\delta }_{j}{IND}_{j}+\epsilon$$

5 $$R=\alpha +{\beta }_{1}SC\_adjusted+{\beta }_{2}LnAsset+{\beta }_{3}BM+{\beta }_{1}Leverage+{\sum }_{j}{\delta }_{j}{IND}_{j}+\epsilon$$

where *j* indexes each stock. *R* is the cumulative return (%) over the 3- and 5-day event window. *R* can be *R*[− 1,1], *R*[− 2,2], $R\mathrm{^{\prime}}$ [− 1,1], $R\mathrm{^{\prime}}$[− 2,2], $R\mathrm{^{\prime}}\mathrm{^{\prime}}$[− 1,1] or $R\mathrm{^{\prime}}\mathrm{^{\prime}}$[− 2,2]. Each *IND*_*j*_ denotes various industry dummies. We test our regression model using both *SI/SC* $\mathrm{and }SI\_adjusted$/ $SC\_adjusted$. Statistically, *SI/SC* $\mathrm{and }SI\_adjusted$/$SC\_adjusted$ generated identical coefficients for all variables except for the industry dummies and the constant terms. Therefore, we only report the results for $SI\_adjusted$/$SC\_adjusted$ in our tables.

## Results

**Fig. 3:**
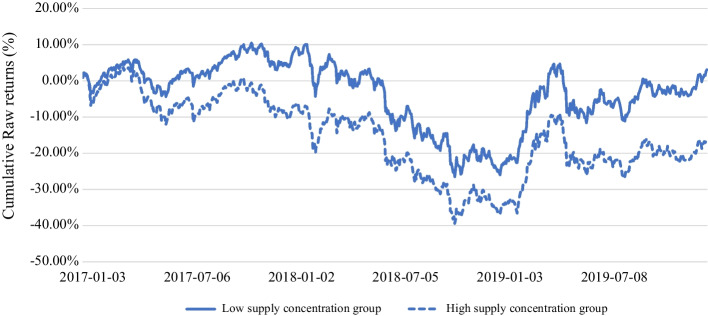
Long-term Portfolio Performance (High vs Low Supplier Concentration Groups). Cumulative raw return for industry neutral high vs low supplier concentration groups over time during Jan 1, 2017 and Dec 31, 2019: This figure plots the cumulative raw return for industry neutral high vs low supplier concentration groups trend evolving over time. At the beginning of each year, we sort stocks into high vs low portfolios based on their sample median supplier concentration scores and track their cumulative raw return in the following year. The portfolios are adjusted every 12 months and weighted by market values of stocks

Before examining the stock market effect of supplier concentration, we explore whether an investment portfolio with lower SC using an industry-neutral classification scheme can generate better cumulative returns during our studied period between 2017 and 2019. Figure 3 provides supportive evidence by plotting industry-neutral, annually re-balanced portfolios constructed using SC scores from January 2017 to December 2019. Industry neutrality is provided by identifying high- or low-SC stocks for each industry to construct the high-SC and low-SC investment portfolios. This classification scheme allows an equal number of stocks for each industry to be presented in the two portfolios, making sure that their superior or inferior investment returns are not the results of industry acentric factors. These industry-neutral portfolios are weighted by the market values of stocks. An interesting observation is that the low-SC portfolio remains consistently greater than that of the high-SC portfolio since the beginning of January 2017. The differential cumulative raw returns for the two groups at the end of 2017, 2018, and 2019 are 14.30%, 11.47%, and 19.20%, respectively. These figures imply that, under normal market conditions, an industry-neutral SC-based investment strategy allows an investor to earn a substantially higher return in the China stock market.

Table 5 displays the correlation coefficients matrix for the variables in our study. As expected, the median-adjusted variables measuring supplier concentration, SI_adjusted, and SC_adjusted, are positively correlated with a correlation coefficient equals to 0.770. SI_adjusted and SC_adjusted are also negatively correlated with cumulative raw returns. All the pair-wise correlation coefficients are lower than 1.

**Table 5:** Correlation matrix

	(a)	(b)	(c)	(d)	(e)	(f)	(g)	(h)	(i)	(j)
(a)R[− 1, 1]	1									
(b)R[− 2, 2]	0.937 (< .0001)	1								
(c)SI	− 0.030 (0.180)	− 0.025 (0.268)	1							
(d)SI_adjusted	− 0.046 (0.042)	− 0.045 (0.045)	0.968 (< .0001)	1						
(e)SC	− 0.032 (0.151)	− 0.034 (0.134)	0.779 (< .0001)	0.762 (< .0001)	1					
(f)SC_adjusted	− 0.035 (0.121)	− 0.038 (0.098)	0.766 (< .0001)	0.770 (< .0001)	0.996 (< .0001)	1				
(g)LnAsset	0.030 (0.271)	0.011 (0.764)	− 0.189 (< .0001)	− 0.197 (< .0001)	− 0.046 (0.003)	− 0.055 (0.002)	1			
(h)BM	− 0.121 (0.003)	− 0.089 (0.004)	− 0.309 (< .0001)	− 0.198 (< .0001)	− 0.213 (< .0001)	0.243 (< .0001)	0.246 (< .0001)	1		
(i)Leverage	− 0.060 (0.008)	− 0.072 (0.002)	− 0.150 (< .0001)	− 0.135 (< .0001)	− 0.058 (0.010)	− 0.056 (0.013)	0.248 (< .0001)	0.288 (< .0001)	1	
(j)Rev_Disclose	0.026 (0.243)	0.019 (0.401)	− 0.017 (0.443)	− 0.022 (0.323)	− 0.023 (0.307)	− 0.024 (0.285)	0.031 (0.165)	0.039 (0.083)	0.029 (0.192)	1

Now we test for the effects of supplier disclosure on stock returns under COVID-19. In Panel A (first four columns) of Table 6, we find that the independent variable SI_adjusted is negatively and significantly related to cumulative returns for both 3-day and 5-day event windows, indicating that firms with a higher degree of supplier disclosure have higher stock price declines during the COVID-19 pandemic. These results indicate that lower supplier disclosure might offer investors some protection against downside risk during this pandemic. These results hold for all models for the total sample and the secondary sector. As the supply chain relationship is more relevant for manufacturing firms than for primary and tertiary sectors. Therefore, we will focus on the secondary sector for our remaining analysis. It should also be noted that SI and industrially median-adjusted SI have the same estimated coefficients and *p*-values, and median adjustment only changes the estimated coefficients of constant terms and industry dummies. This finding suggests that supplier disclosure appears to have an impact on the stock price effect during the crisis. However, as almost all firms (except two) only disclose top 5 suppliers, the SI variable and the SC variable are highly correlated (as indicated in the correlation matrix in Table 5), leading to the conclusion that we can focus more on SC for the rest of the paper as its role is more documented in the literature.

**Table 6:** Regression analysis for SI_adjusted

Variables	*R*[− 1, 1] All	*R*[− 2, 2] All	*R*[− 1, 1] Secondary	*R*[− 2, 2] Secondary	*R*[− 1, 1] All	*R*[− 2, 2] All	${R}^{^{\prime}}$[− 1, 1] All	${R}^{^{\prime}}$[− 2, 2] All
**Panel A**: cumulative raw return (*R*) with SI_adjusted as main variable					**Panel B**: cumulative raw return (*R*) and cumulative market-adjusted return (${R}^{^{\prime}}$) with SI_adjusted, RevDis_dummy, and SI_adjusted x RevDis_dummy as main variables			
**SI_adjusted**	− 0.014 ** (− 2.31)	− 0.019** (− 2.31)	− 0.018** (− 2.51)	− 0.023** (− 2.25)	− 0.020** (− 2.35)	− 0.025 (− 1.43)	− 0.024 (− 0.68)	− 0.032 (− 0.73)
RevDis_dummy					0.532 (1.32)	0.412 (0.81)	0.714* (1.89)	0.431 (1.25)
SI_adjusted x RevDis_dummy					− 0.027 (− 1.44)	− 0.029 (− 1.12)	− 0.040 (− 1.17)	− 0.061 (− 1.27)
LnAsset	1.524*** (3.76)	1.897*** (3.22)	1.679** (2.56)	2.011** (2.35)	1.865*** (3.79)	2.124*** (3.03)	1.898** (2.16)	2.267 (1.65)
BM	− 7.023*** (− 3.56)	− 6.573*** (− 3.46)	− 7.347*** (− 3.23)	− 4.259*** (− 3.25)	− 7.276*** (− 3.21)	− 6.031*** (− 3.28)	7.986*** (− 3.56)	5.147*** (− 3.33)
Leverage	− 3.765 (0.62)	− 3.535 (0.04)	− 4.098 (0.06)	− 4.572 (− 0.67)	− 3.589 (0.61)	− 3.352 (0.05)	− 4.572 (0.09)	− 4.375 (− 0.75)
Constant	− 3.654 (0.77)	− 2.478 (0.81)	− 2.179 (0.43)	− 2.017 (0.74)	− 3.089 (0.75)	− 2.243 (0.78)	− 2.135 (0.51)	− 1.986 (0.58)
Location FE	Yes	Yes	Yes	Yes	Yes	Yes	Yes	Yes
Observations	1965	1965	1558	1558	1965	1965	1965	1965
R-squared	0.154	0.134	0.124	0.137	0.176	0.145	0.165	0.147

Cumulative raw return (*R*) and cumulative market-adjusted return (${R}^{^{\prime}}$) as DV. All the regressions include controls variables and location fixed effects. *, **, and *** indicate significance at the 10%, 5%, and 1% levels, respectively. All reported t statistics are based on standard errors adjusted for clustering at the industry level

We further examine the incremental effect of reverse disclosure. As this information is gathered through third-party public sources instead of firms’ annual reports, we want to explore how the investors may incorporate this additional information into the stock market valuation. In this case, we construct a dummy variable *RevDis_dummy* to identify if there is reversely disclosed supplier information for a certain firm (which is not covered by the forward disclosure). In Panel B (last four columns) of Table 6, we examine the effect of supplier information from reverse disclosure by taking both cumulative raw returns and cumulative market-adjusted returns over the 3-day and 5-day windows as dependent variables. The results show that both *RevDis_dummy* and its cross-product term with SI_adjusted have insignificant effects on returns. Therefore, in our subsequent analysis, we do not consider the separate role of reversely disclosed supplier information. We will use forward and reverse data to estimate the median-adjusted form of SC to measure supplier concentration.

Table 7 presents the main results of the effect of supplier concentration. For the first four columns, the dependent variables are *R*[− 1,1] and *R*[− 2,2] for all firms and the secondary sector over the 3- and 5- trading day windows around the Wuhan lockdown. We regress the cumulative raw returns on the SC scores, after controlling for leverage, book-to-market, and firm size. We also include 25 industry dummies based on the sector code from SWS Research.

**Table 7:** Regression analysis for SC_adjusted

Variables	*R*[− 1, 1] All	*R*[− 2, 2] All	*R*[− 1, 1] Secondary	*R*[− 2, 2] Secondary	${R}^{{{\prime}}{{\prime}}}$[− 1, 1] All	${R}^{{{\prime}}{{\prime}}}$[− 2, 2] All	${R}^{{{\prime}}{{\prime}}}$[− 1, 1] Secondary	${R}^{{{\prime}}{{\prime}}}$[− 2, 2] Secondary	${R}^{{{\prime}}{{\prime}}}$[− 2, 100] All	${R}^{{{\prime}}{{\prime}}}$[− 2, 100] Secondary
**SC_adjusted**	− 3.001** (− 2.04)	− 3.923 (− 2.14)	− 2.813** (− 2.27)	− 4.098** (− 2.17)	− 4.125*** (− 3.04)	− 5.364** (− 2.35)	− 7.254*** (− 4.58)	− 8.012*** (− 4.41)	− 10.213** (− 2.21)	− 16.354** (− 2.26)
LnAsset	1.408*** (4.22)	51.582*** (4.26)	1.674*** (4.14)	1.915*** (3.70)	1.013*** (3.71)	1.214*** (4.01)	1.243*** (4.59)	1.565*** (4.57)	1.653*** (3.98)	2.017*** (4.49)
BM	− 5.177*** (− 6.52)	− 5.834*** (− 5.89)	− 4.615*** (− 5.11)	− 5.026** (− 4.19)	− 4.479*** (− 4.22)	− 5.932*** (− 4.32)	− 5.232*** (− 3.78)	− 6.458*** (− 3.27)	− 13.503*** (− 4.61)	− 16.519*** (− 4.57)
Leverage	− 4.845* (− 1.97)	− 6.847*** (− 6.63)	− 6.168** (− 2.23)	− 8.303*** (− 6.37)	− 2.113 (− 1.44)	− 3.786** (− 2.34)	− 3.259* (− 1.74)	− 4.568** (− 2.56)	− 13.335 (− 1.43)	− 15.636* (− 1.66)
Constant	− 5.058** (− 2.22)	− 2.698 (− 1.48)	− 4.776* (− 1.79)	− 2.819 (− 1.31)	1.987 (0.49)	2.429 (1.26)	1.738 (0.56)	2.286 (1.19)	20.453*** (4.25)	22.135*** (4.97)
Location FE	Yes	Yes	Yes	Yes	Yes	Yes	Yes	Yes	Yes	Yes
Observations	1965	1965	1558	1558	1965	1965	1558	1558	1965	1558
R-squared	0.131	0.114	0.147	0.129	0.165	0.146	0.171	0.150	0.099	0.123

Raw Return cumulative (*R*) and cumulative abnormal return (${R}^{{{\prime}}{{\prime}}})$ based on CAPM for short- and medium-term event windows as DV and SC_adjusted as IV. All of the regressions include control variables and location fixed effects. *, **, and *** indicate significance at the 10%, 5%, and 1% levels, respectively. All reported t statistics are based on standard errors adjusted for clustering at the industry level

We find the independent variable SC_adjusted is negatively and significantly related to the short-term cumulative raw return for all models for the total sample and the secondary sector. This suggests firms with a higher degree of supplier concentration have higher stock price declines during the COVID-19 pandemic. We find that the significant effect of the industrially median-adjusted variable (i.e. SC_adjusted) on returns still exists in the secondary sector. The next four columns (i.e. columns 5–8) of Table 7 reports the regression results when ${R}^{{{\prime}}{{\prime}}}$[− 1, 1] and ${R}^{{{\prime}}{{\prime}}}$[− 2, 2] are dependent variables and other settings are the same as those in the earlier columns. The results are similar and show that the significant effect of SC_adjusted on cumulative abnormal returns still exists for all firms in our sample and firms in the secondary sector.

Afterward, we examine whether the negative results for supplier concentration may hold when we expand the event window to an extended period. The last two columns of Table 7 report the regression results using cumulative abnormal returns between 22 January and 30 June 2020, denoted by ${R}^{{{\prime}}{{\prime}}}$[− 2, 100], on SC_sdjusted, while other settings are the same as those models in earlier columns of Table 7. It shows that the significant effect of SC_sdjusted on the cumulative abnormal return for this medium-term event window still exists.

We conducted various robustness tests on our main results.^14^ At the time of our data purchase in early 2020, 1073 listed firms had not reported the supplier data, which substantially reduced our sample size. During the revision process up till early 2021, these firms have eventually filed the data with CSMAR. We now added back the data with an expanded sample of 2302 and reconducted the main regression analysis for Table 7. 15 The results remain the same and are reported in the Appendix F.

In addition, we treat cumulative market-adjusted return, as an alternative return measure. The results indicate that the significant effects of SI_adjusted and SC_sdjusted on returns still exist for all firms in the sample and the firms in the secondary sector. Second, we regress the cumulative raw return in 2019 on SI_adjusted and SC_sdjusted in 2018, after controlling for leverage, book-to-market, firm size, and industry. These results show that SI_adjusted in 2018 has no significant effect on the cumulative raw return in 2019 for all firms in the sample as well as the firms in each of the three sectors. But SC_sdjusted in 2018 only has a significant effect on the cumulative raw return in 2019 for all firms in the sample at a 10% significance level. The results indicate that no significant effect of supplier concentration on stock returns can be found in a normal period.

Next, we conduct a matched-pair comparison (controlled for industry, market-to-book, and firm size) as a benchmark for a two-sample T-test to confirm that the CAR differences between high and low SC groups are not affected by industry, growth, and size. Specifically, we use industry, size, and market-to-book to identify matching firms to recompute the abnormal return differences between high SC concentration and low concentration samples. We first control the matching pair using firms within the same industry for the high and low supplier concentration (SC) samples. High SC must be within the top 25% while low SC is within the bottom 25% of the SC values. The matching criteria of size and market-to-book are ± 30% for the matched pair. There is no repeating use of the matching firms so each matched pair of high and low SC firms is a unique combination of firms. In short, the sample returns are computed based on industry-neutral high vs low supplier concentration groups after controlling for size and market-to-book. Appendix B reports the high supply chain concentration (SC) and low SC sub-samples with the controls. The result shows that the high SC sub-sample suffers from a significantly lower return (i.e. more negative CAR) relative to that of the low SC sub-sample.

Furthermore, using a regression format, Appendix C tests a difference in abnormal return comparing the hardest hit provinces with the others. In Mainland China, a lockdown of the city means that social distancing policy is strictly imposed. In other words, residents including employees of firms living in the lockdown city are not allowed to leave their homes or subdivisions. Supply chain administrative process initiates at headquarters. Thus, when the headquarters are in hardest hit provinces, the supply chain can be broken at where it begins because office lockdown prevents purchase orders to be initiated as most of the procurement process in Mainland China is not completely computerized. Therefore, the employees in headquarters basically cannot function at all to conduct business even their suppliers may be away and are not affected by the lockdown. In addition, there is a good chance that some suppliers will be in the same lockdown city. Therefore, it is possible that higher SC firms located in the hardest hit provinces suffering lockdown would have more negative stock price reaction because of the reasons above. Appendix C shows our regression finding based on hardest hit sub-sample vs other provinces. The SC coefficient is negatively significant for the hardest hit sub-sample but not significant for the other provinces. The finding shows that the location effect is significant, meaning that firms with higher SC and headquarters in the lockdown provinces suffer stronger in terms of stock price reactions in our event study. For firms with headquarters in further away provinces, SC has no effect on the event period abnormal returns. This result demonstrates that with a more restrictive sample control comparison and regression analysis, our main results still hold.

Now, we run our regression by showing that our supplier concentration variable (SC_adjusted) provides additional explanation power beyond business risk and profitability. Since there is no standard variable to proxy business risk and earnings volatility is indeed an appropriate measure of business risk, we choose the standard deviation of EPS to measure business risk. Also, ROA is added to measure profitability. Finally, our existing regression model has already included a leverage variable which can be viewed as a financial constraint proxy. In short, we have added variables for business risk, profitability, investment, and cash to see the effect.^16^ Appendix E reports the result by adding these controls to the existing regression model in Table 7 (our main result). As indicated in all columns using both (*R*) and (*R*″)_,_ the SC_adjusted variable coefficients remain negatively significant in all cases. This result supports our main hypothesis that supplier concentration does matter after controlling for these additional variables.^17^

Finally, we examine if productivity of firms jointly affects degree of supplier concentration and stock market performances, as it is also possible that less productive firms might have had difficulties in diversifying their suppliers, which could result in higher supplier concentration and lower stock market performances. To explore this alternative explanation of whether less productive firms might have had difficulties in diversifying their suppliers, we design Appendices G to show the results.^18^ Appendix G compares productivity (measured by ratio of annual operating revenue to number of employees at year end) for 2016–2019. The ratios are statically the same for high and low SC groups as all the two-sample t-tests are not significant. In fact, nominally speaking, the high SC groups actually have slightly better productivity ratios throughout the period, which is opposite to the proposed alternative explanation.

Finally, we addressed the issue of whether productivity has a differential effect during the event. We conducted event study to estimate the *R*″[− 1, 1] and *R*″[− 2, 2] for the high vs low productivity groups. In Appendix H, the differential abnormal returns for these two subsamples are not significantly different from zero, indicating that productivity is not a determinant for announcement effect for our COVID-19 event. As our Table 7 clearly demonstrates that SC has a significant impact to abnormal returns for the COVID-19 event, we can safely conclude that our event study result for SC is not related to productivity.

## Discussion and conclusion

The recent literature on supply chain viability and resilience have pointed out the importance of understanding the quantitative drivers of resilience and risk mitigation tools, especially under disruptions such as COVID-19 (Hosseini et al. 2019; Ivanov and Dolgui 2020; Ivanov and Das 2020). The COVID-19 global pandemic creates new challenges for supply chain viability and to maintain resilience, adaptability, and sustainability. One efficient and commonly used measure to evaluate the success of supply chain coping mechanisms for listed firms is to observe how the stock market investors reacted when there exists an unexpected disruption.

We employ a unique set of supply chain data provided by MioTech’s AI engine which covers all listed firms in China. Our initial finding shows that an industry-neutral, annually re-balanced portfolio with stocks of low supplier concentration outperform a stock portfolio of high supplier concentration by a cumulative return of 19.20% during the period between 2017 and 2019. Such preliminary evidence of superior investment strategies using supplier concentration deserves further research attention, but it is beyond the scope of our study.

Our main research question is how concentrated suppliers may negatively affect stock valuation caused by a potential supply chain deterioration when investors react to accumulated and negative news shock after the Wuhan lockdown as a result of the COVID-19 pandemic. Madsen (2017) shows that investor attention to customer–supplier disclosure increases when suppliers’ information arrival is expected. The city lockdowns in China and the social distancing policy to control the COVID-19 infected cases contain negative information shock on potential disruption of the supply chain. The corresponding investor attention to this negative supply chain information should lead to a price devaluation for firms with high supplier concentration risk.

We control for the potential effect of supply chain deterioration by geographical location (i.e. Hubei province where Wuhan is located and the top 5 hardest-hit provinces) and employ both forward and reverse disclosure data. We also construct short-term windows (i.e. [− 1, 1] and [− 2, 2]) and a medium-term window [− 2, 100] around the periods after the announcement of the Wuhan lockdown. All findings indicate that a higher supplier concentration leads to a stronger price drop. This result is consistent with the conjecture that market investors worry about the negative effect of supply chain deterioration on firms with high concentration risks. Therefore, investors react accordingly when they first get a chance at the opening of the China stock market after the Chinese New Year holidays after the Wuhan lockdown.

Our study has shed insight into firms’ top management to seriously consider supplier diversification to prevent unexpected supply chain disruption. While various literature has documented certain advantages financially for supplier concentration, such advantages should be viewed in a relation to the downside risk as indicated in our findings. On the other hand, investment managers also need to pay attention to supply chain data and their effect on stock valuation. Our study indicates that, during the normal period, lower supply concentration can generate superior returns for an investment portfolio. In addition, lower supplier concentration can also reduce stock price decline during unexpected nationwide crisis.

Since data on location of suppliers does not exist and is impossible to collect due to the fact that most of the suppliers of our sample firms are not listed, with no feasible method to collect such information,^19^ our analysis examines the location of headquarters and its effects on abnormal returns. While this research does not address the effects of location of suppliers on the robustness of supply chains, we believe that headquarters location can be useful to explore supply chain deterioration due to lockdown. The rationale is that supply chain administrative process initiates at headquarters. Thus, when the headquarters are in hardest hit provinces, the supply chain can be broken at where it begins because office lockdown prevents purchase orders to be initiated as most of the procurement process in Mainland China is not completely computerized.

In fact, the literature recognizes that location plays an important role in diversifying risks of a supply chain and has been recognized as one the remarkable sources of risk in supply chains (Tang and Musa 2011; Mukherjee and Padhi 2022). Recent studies address this issue by developing analytical models, experimental studies, and survey studies. For example, Deane et al. (2009) develop a multi-criteria optimization model to show that global sourcing can enhance the performance of supply chains, but will also bring potential devastating effects of supply disruptions. Zhang et al. (2016) examine the risk-pooling effect and economic scale of location model while controlling for the supply disruption risk, Habermann et al. (2015) evaluate if dispersion of supply chain partners can help to reduce disruption risk by survey analysis. They find that co-location with suppliers, instead of dispersion, tends to mitigate disruption risk because of deeper collaboration with close suppliers. However, no research has been conducted by analyzing the secondary location data since such data usually is not available. In MioTech and CSMAR database, supplier’s location information is also not available. Although one can obtain the location information of a supplier if it is also a listed company, most suppliers of listed companies are not listed. Further study on the impacts of suppliers’ location on stock prices over the trading day windows could be conducted if associated data can be identified.

## Limitation and future research

Future research directions can be numerous. First, previous studies suggest that customer concentration may also post a negative impact on asset pricing. How would the firms with various degrees of SC have differed when their customers are concentrated or fragmented? Customer concentration can be integrated in future research to examine whether customers and supplier concentration may have an interactive effect on valuation. Second, when annual financial data are available for 2020, the effect of supplier concentration on firms’ financial performance and operational performance after the outbreak of COVID-19 could also be a focus. Third, further research can focus on how government financial stimuli may benefit firms with supply chain deterioration. In fact, further studies may collect data from other industrial-oriented economies such as the United States, Germany, Japan, and South Korea, to examine the global effect of supplier concentration on stock returns during the COVID-19 crisis.

In addition, do firms in China in general switch suppliers as a practice under normal conditions? Appendix D lists the supplier changes for the 2019 sample. Owing to missing data for the comparison procedure, we can only compute the supplier changes from 2018 to 2019 for a total of 1087 firms. Appendix D shows the distribution of supplier changes.^20^ 40.2% of firms made no changes in the top 5 suppliers. The remaining 59.8% of firms changed one to all 5 suppliers. In other words, changing top 5 suppliers appear to be a relatively common practice in this preliminary analysis. A more in-depth analysis (which is beyond the scope of our current study) for future research can be considered.^21^

Finally, we have to admit that, we do not find strong value in enhancing significance of our finding by adding reverse disclosure data. However, we will not know if this is the case unless we spent the effort to conduct the analysis. As indicated in Table 6, the *RevDis_dummy* is only marginally significant in one of the four models, casting doubt to the added value of reverse disclosure in our analysis. Therefore, future research may not need to spend extra resources to collect data from reverse disclosure for a similar analysis when dealing with China firms.

## Data Availability

The data that support the findings of this study are available from MioTech and Datastream but restrictions apply to the availability of these data, which were used under license for the current study, and so are not publicly available. Data are however available from the authors upon reasonable request and with permission of MioTech and Datastream.

## Appendix A

See Table 8.

**Table 8:** Variable definition and data sources

Variable	Definition	Data source
Panel A: Dependent and test variables		
*SI*	Supplier index, calculated as the sum of a firm’s percentage of supply from the top five suppliers and the percentage of supply amounts from reverse disclosure in the firm’s annual report	MioTech
*SC*	Supplier concentration, calculated as the sum of the squared weight of the top five suppliers’ amount	MioTech
*SI_adjusted* *(SC_adjusted)*	The median adjusted *SI* (*SC*)by subtracting the median of *SI* (*SC*)of each industry from *SI* (*SC*)	MioTech
*R*[− 1,1] *(R*[− 2,2]*)*	The cumulative raw return in percentage over the three (five)-trading day window of 23 Jan– 4 Feb 2020 (22 Jan– 5 Feb 2020) after Wuhan lockdown during the COVID-19 outbreak	CSMAR
$R\mathrm{^{\prime}}$[− 1,1] *(*$R\mathrm{^{\prime}}$[− 2,2])	The cumulative Market-adjusted Return for Jan 23 – Feb 4 2020 (22 Jan – 5 Feb 2020) calculated by subtracting the market return from the raw return	CSMAR
$R\mathrm{^{\prime}}\mathrm{^{\prime}}$[− 1,1] *(*$R\mathrm{^{\prime}}\mathrm{^{\prime}}$[− 2,2])	The cumulative abnormal return for 23 Jan– 4 Feb 2020 (22 Jan – 5 Feb 2020) calculated by subtracting the expected return based on CAPM model from the raw return, while the beta estimation of CAPM is over 200 trading days i.e. 4 March 2019 – 23 December 2019	CSMAR
${R}^{{{\prime}}{{\prime}}}$[− 2, 100]	The cumulative abnormal return for 22 Jan – 30 June 2020 calculated by subtracting the expected return based on CAPM model from the raw return, while the beta estimation of CAPM is over 200 trading days i.e. 4 March 2019 – 23 December 2019	CSMAR
*LnAsset*	The logarithm of total assets in RMB 10 billion	CSMAR
*BM*	The ratio of book value per share to the stock price per share	CSMAR
*Leverage*	The ratio of total liability to total assets	CSMAR
*Invest/Asset*	The ratio of investment to total assets	CSMAR
*Cash/Asset*	The ratio of cash and cash equivalents to total assets	CSMAR
*RevDis_dummy*	The dummy variable to represent if there is reversely disclosed supply information for a certain firm, which is not covered by forward disclosure	MioTech

## Appendix B

See Table 9.

**Table 9:** Matched sample comparison for high SC vs low SC

	(1)	(2)	Dif. (2) - (1)
	High supplier concentration	Low supplier concentration	
Three-day cumulative raw returns	(N = 504) − 12.962%	(N = 504) − 11.703%	− 1.259% (− 2.23)**
Five-day cumulative raw returns	(N = 504) − 10.355%	(N = 504) − 8.379%	− 1.976% (− 2.93)***

Two sample t-Test results: Three-day cumulative raw returns over the three-trading day window (Jan 23 – Feb 4 2020) and the five-trading day window (Jan 22 – Feb 5 2020) after Wuhan lockdown. The sample returns are computed based on industry neutral high vs low supplier concentration groups after controlling for size and market-to-book. High SC is the top 25% and low SC is the bottom 25% of the SC values. The matching criteria of size and market-to-book are ± 30%. There is no repeating use of the matching firms so each matched pair of high and low SC firms is a unique pair

## Appendix C

See Table 10.

**Table 10:** Location effect

Variables	${R}^{{{\prime}}{{\prime}}}$[− 1, 1] Hardest hit provinces and close neighbors	${R}^{{{\prime}}{{\prime}}}$[− 2, 2] Hardest hit provinces and close neighbors	${R}^{{{\prime}}{{\prime}}}$[− 1, 1] Other provinces	${R}^{{{\prime}}{{\prime}}}$[− 2, 2] Other provinces
**SC_adjusted**	− 6.875** (− 2.12)	− 7.538** (− 2.24)	− 2.431 (− 1.34)	− 3.894 (− 1.42)
LnAsset	1.263*** (2.41)	1.382*** (2.89)	0.826 (1.54)	1.098 (0.34)
BM	− 3.625*** (− 3.43)	− 4.353*** (− 2.90)	− 5.498** (− 2.04)	− 6.764*** (− 2.78)
Leverage	− 3.037 (− 1.45)	− 4.984** (2.23)	− 1.101 (1.56)	− 3.010 (− 0.30)
Constant	2.608 (0.91)	1.045 (− 1.29)	1.513** (− 2.17)	2.728*** (− 4.93)
Observations	1028	1028	937	937
R-squared	0.169	0.153	0.208	0.187

Cumulative abnormal return (${R}^{{{\prime}}{{\prime}}})$ based on CAPM for short- and medium-term event windows as DV and SC_adjusted as IV for hardest hit provinces (with their close neighbors) and other provinces. All of the regressions include control variables. *, **, and *** indicate significance at the 10%, 5%, and 1% levels, respectively. The top five hardest hit provinces are Hubei, Guangdong, Henan, Zhejiang, Hunan (Source: China Data Lab, 2020, “China COVID-19 Daily Cases with Basemap”. https://doi.org/10.7910/DVN/MR5IJN, Harvard Dataverse, V32). And the close neighbors of them include Anhui, Chongqing, Jiangxi, Shaanxi, Fujian, Guangxi. All reported t statistics are based on standard errors adjusted for clustering at the industry level

## Appendix D

See Table 11

**Table 11:** 2018–19 suppliers’ changes distribution

No change	Changed 1 in top 5	Changed 2 in top 5	Changed 3 in top 5	Changed 4 in top 5	Changed 5 in top 5	Sub-total
437 (40.20%)	213 (19.60%)	141 (12.97%)	124 (11.41%)	91 (8.37%)	81 (7.45%)	1087 (100%)
Sample firms with top 5 suppliers transaction amount reported in 2019 but some top suppliers’ real names not disclosed in either 2018 or 2019 or both*						1320
2019 sample size before removing financial and utility firms						2407
2019 financial and utility firms removed						105
2019 sample size						2302

^*^The undisclosed supplier means that they have the figures for top 5, but the name is like "Supplier A", "Supplier B", "Supplier C", etc. As such, in those cases, one cannot compare whether it has changed

.

## Appendix E

See Table 12

**Table 12:** Robustness Analysis for Table 5 by Adding Additional Controls for Business Risk (Standard deviation of EPS), Profitability (ROA), Ratio of total security investment to total asset and Ratio of Cash and Cash Equivalents to total asset)

Variables	*R*[− 1, 1] All	*R*[− 2, 2] All	*R*[− 1, 1] Secondary	*R*[− 2, 2] Secondary
**SC_adjusted**	− 3.437** (− 2.14)	− 4.531** (− 2.40)	− 3.044** (− 2.58)	− 4.764** (− 2.21)
LnAsset	1.215*** (3.60)	1.380*** (3.81)	1.435** (2.89)	1.660*** (3.79)
BM	− 4.617*** (− 5.51)	− 5.233*** (− 5.14)	− 4.018*** (− 3.90)	− 4.363*** (− 4.91)
Leverage	− 4.977* (− 1.89)	− 7.164*** (− 5.94)	− 6.216** (− 2.18)	− 8.592*** (− 5.68)
EPS_std	0.887 (1.17)	− 0.535 (0.91)	1.301 (1.62)	0.950 (1.48)
ROA	0.171*** (5.05)	0.207*** (3.39)	0.177*** (4.21)	0.216*** (3.03)
Invest/Asset	− 6.341 (− 0.07)	− 13.477 (− 0.12)	16.668 (0.12)	17.209 (0.12)
Cash/Asset	− 0.439 (− 0.24)	− 1.996 (− 0.85)	0.056 (0.02)	− 1.636 (− 0.56)
Constant	− 5.324** (− 2.65)	− 2.453 (− 1.24)	− 5.260** (− 2.25)	− 2.768 (− 1.14)
Location FE	Yes	Yes	Yes	Yes
Observations	1965	1965	1558	1558
R-squared	0.142	0.124	0.159	0.139

Variables	${R}^{{{\prime}}{{\prime}}}$[− 1, 1] All	${R}^{{{\prime}}{{\prime}}}$[− 2, 2] All	${R}^{{{\prime}}{{\prime}}}$[− 1, 1] Secondary	${R}^{{{\prime}}{{\prime}}}$[− 2, 2] Secondary
**SC_adjusted**	− 5.578** (− 2.32)	− 6.879** (− 2.21)	− 9.012*** (− 4.24)	− 10.031*** (− 4.15)
LnAsset	1.211*** (3.44)	1.312*** (3.77)	1.512*** (4.79)	1.735*** (4.44)
BM	− 5.133*** (− 4.10)	− 6.015*** (− 3.99)	− 7.324*** (− 3.93)	− 7.995*** (− 3.56)
Leverage	− 1.897 (− 1.52)	− 2.783** (− 2.37)	− 2.457 (− 1.46)	− 3.587** (− 2.46)
EPS_std	0.745 (0.93)	0.459 (0.78)	0.690 (1.12)	0.398 (0.85)
ROA	0.192*** (4.11)	0.241*** (4.26)	0.189*** (3.97)	0.297*** (4.01)
Invest/Asset	3.845 (1.01)	5.023 (1.08)	5.956 (1.09)	6.586 (1.13)
Cash/Asset	0.231 (0.15)	− 1.247 (− 0.65)	− 0.132 (− 0.11)	− 1.785 (− 0.89)
Constant	2.341 (0.98)	2.797 (1.43)	2.586 (1.03)	3.213 (1.09)
Location FE	Yes	Yes	Yes	Yes
Observations	1965	1965	1558	1558
R-squared	0.178	0.157	0.168	0.168

Raw Return cumulative (*R*) and cumulative abnormal return (${R}^{{{\prime}}{{\prime}}})$ based on CAPM for short- and medium-term event windows as DV and SC_adjusted as IV. All of the regressions include control variables (Logged Total assets, Book to market ratio, Ratio of total liability to total asset, ROA, Standard deviation of EPS, Ratio of investment to total asset and Ratio of cash and cash equivalents to total asset), location fixed effects. *, **, and *** indicate significance at the 10%, 5%, and 1% levels, respectively. All reported t statistics are based on standard errors adjusted for clustering at the industry level

.

## Appendix F

See Table 13

**Table 13:** Regression analysis for SC_adjusted

Variables	${R}^{{{\prime}}{{\prime}}}$[− 1, 1] All	${R}^{{{\prime}}{{\prime}}}$[− 2, 2] All	${R}^{{{\prime}}{{\prime}}}$[− 1, 1] Secondary	${R}^{{{\prime}}{{\prime}}}$[− 2, 2] Secondary	${R}^{{{\prime}}{{\prime}}}$[− 2, 100] All	${R}^{{{\prime}}{{\prime}}}$[− 2, 100] Secondary
**SC_adjusted**	− 8.462*** (− 2.97)	− 8.611** (− 2.57)	− 10.403*** (− 5.53)	− 10.177*** (− 4.05)	− 13.809** (− 2.11)	− 19.288** (− 2.30)
LnAsset	1.212*** (4.67)	1.342*** (3.90)	1.521*** (5.54)	1.719*** (4.53)	2.013*** (4.67)	2.539*** (6.37)
BM	− 5.769*** (4.67)	− 6.335*** (− 4.34)	− 5.805*** (− 4.02)	− 6.080*** (− 3.16)	− 17.209*** (− 4.82)	− 19.666*** (− 4.89)
Leverage	− 3.071 (− 1.44)	− 5.485** (− 2.54)	− 4.216* (− 1.77)	− 6.763** (− 2.82)	− 16.314 (− 1.54)	− 17.741* (− 1.78)
Constant	2.299 (0.59)	3.233 (0.96)	2.243 (0.48)	2.787 (0.71)	23.888*** (5.25)	27.188*** (5.29)
Location FE	Yes	Yes	Yes	Yes	Yes	Yes
Observations	2302	2302	1844	1844	2302	1844
R-squared	0.149	0.138	0.161	0.142	0.098	0.115

Cumulative abnormal return (${R}^{{{\prime}}{{\prime}}})$ based on CAPM for short- and medium-term event windows as DV and SC_adjusted as IV. All of the regressions include control variables, location effects. *, **, and *** indicate significance at the 10%, 5%, and 1% levels, respectively. All reported t statistics are based on standard errors adjusted for clustering at the industry level

.

## Appendix G

See Table 14

**Table 14:** Firm productivity (measured by ratio of operating revenue of the year to number of employees at the end of the year) divided by High and Low Supplier Concentration Groups

	Mean of the ratio of operating revenue of the year to number of employees at the end of the year ( unit for operating revenue: CNY1 million)			
	2016	2017	2018	2019
Low concentration group	0.643	0.708	0.776	0.818
High concentration group	0.678	0.737	0.785	0.859
Two-sample t- test	− 0.035 (1.55)	− 0.029 (0.49)	− 0.009 (0.827)	− 0.041(0.184)

.

## Appendix H

See Table 15

**Table 15:** Cumulative abnormal returns (${{\varvec{R}}}^{\boldsymbol{^{\prime}}\boldsymbol{^{\prime}}})$ based on CAPM by productivity groups

	High productivity group	Low productivity group	Difference
${R}^{{{\prime}}{{\prime}}}$[− 1, 1]	− 1.196	− 1.134	− 0.062 (0.17)
${R}^{{{\prime}}{{\prime}}}$[− 2, 2]	− 0.768	− 0.588	0.180 (0.44)

.

## Footnotes

1 “COVID-19 impacts on China’s economy and global supply chains: Recent developments and updated assessments”, *Fung Business Intelligence*, 7 April 2020. https://www.fbicgroup.com/sites/default/files/COVID-19%20impacts_recent%20developments%20and%20updated%20assessments.pdf

2 China Data Lab, 2020, "China COVID-19 Daily Cases with Basemap”. https://doi.org/10.7910/DVN/MR5IJN, Harvard Dataverse, V32.

3 “Covid-19 Impact on China A-Shares’ Supply Chains” V1.0 February 2020, MioTech.

4 While it is interesting to explore the impacts on these industries and in these provinces. Unfortunately, our supply chain data do not go into the details to allow us for such an analysis.

5 Through checking financial news content and stock index pattern before the market closed for the Chinese New Year holidays, no significant sign of market decline is observed.

6 By 23 January 2020, the Chinese government imposed a lockdown in the city of Wuhan (i.e. Wuhan lockdown) to control for the outbreak of COVID-19. Within hours of the Wuhan lockdown, nearby cities of Huanggang and Ezhou also announce travel restrictions and follow similar quarantine measures. Eventually, lockdowns were imposed on 15 other cities in Hubei, affecting about 57 million people. In February 2020, Wenzhou, Zhejiang, implemented a 7-day lockdown and closed most of the highways. By April 2020, the Wuhan lockdown officially comes to an end.

7 Many articles report the continuation of serious economic impact of supply chain disruption due to COVID-19. For instance, Accenture, a global consulting firm, reports that 94% of Fortune 1000 companies are seeing supply chain disruptions from COVID-19 and 75% of companies have had negative or strongly negative impacts on their businesses (https://www.accenture.com/hk-en/insights/consulting/coronavirus-supply-chain-disruption). For more articles, please see: https://www.reuters.com/business/global-markets-supply-pix-2021-11-03/, https://www.morganstanley.com/ideas/supply-chain-disruption-outlook, and https://www.cnbc.com/2022/01/31/china-covid-zero-disrupts-supply-chains-impacts-global-recovery-hsbc-.html

8 PwC China, AmCham China, and AmCham Shanghai. 2020 April. Supply chain strategies under the impact of COVID-19 of large American companies operating in China. Available at: https://www.pwccn.com/en/services/consulting/publications/supply-chain-strategies-under-impact-covid-19-large-american-companies-operating-china.html

9 However, in the subsequent revision, we are able to capture the missing 2019 data and the regression analysis in Table 4 and 5 have increased to 2302.

10 We explore the number of event firms that disclose more than the mandatory requirement of the top 5 suppliers. Only 2 firms have done so in our studied period. Therefore, our disclosure and concentration variables adopt the top 5 data only.

11 Due to limited space, our industry classifications for primary, secondary, and tertiary sectors are not listed here but available upon request.

12 We actually run the regressions using SI and SC as well. However, except for the constant term and the industry control, the regression coefficients for all variables are identical using SI vs SI_adjusted and SC vs SC_adjusted. Therefore, we only show results for the adjusted variables in the paper.

13 We thank two anonymous referees for suggesting location fixed effects in addition to standard errors clustering at the industry level.

14 Due to limited space, our results for all the robustness tests are not listed here but is available upon request.

15 Our initial sample was 1965 for Tables 4 and 5. An anonymous referee raised the issue of possible sample bias and requested us to add back missing supplier data due to delayed annual reports in 2019 as a consequence of COVID-19 after the two rounds of revision, which was 6 months later from the first submission date. We now added back the data with an expanded sample of 2302 and reconducted the main regression analysis in Table 5. The results remain the same and are reported in the Appendices.

16 We thank an anonymous referee for suggesting these additional control variables.

17 We thank an anonymous referee for suggesting this analysis.

18 We thank an anonymous referee for suggesting this analysis.

19 Unfortunately, in both suppliers’ database (MioTech and CSMAR), a large amount of location information of suppliers is not available because suppliers’ names are represented in annual reports by symbols (e.g. 1,2,3… or A,B,C…). Even when suppliers’ names are given, it would still be difficult to obtain the location of most suppliers as they are not listed companies and have limited public information in identifying exact locations of most suppliers.

20 The sample size for 2019 was 1965 firms after removing 97 financial and utility firms, leaving a total of 2062 firms with top supplier transaction amounts reported for calculating SC ratios. However, 1614 out of 2062 firms (before removing 97 financial and utility firms) did not report some of the real names of the top five suppliers, making a comparison with 2018 to calculate a change of supplier during normal period in feasible for these firms. Therefore, we can only compute the supplier changes from 2018 to 2019 for a total of 448 firms.

21 We thank an anonymous referee to suggest this analysis. This statistic is useful to see that there is a regular change of supplier regardless of COVID-19. However, the 2018–2019 supplier change statistic should not be related to our analysis. Our research argument focuses on the disruption of supply chain due to COVID-19 and the market’s corresponding stock price revaluation based on the supplier concentration of the firms. Therefore, we believe that this is a future research issue.
